# Structure of resilience: A Machiavellian contribution or ‘paddle your own canoe’

**DOI:** 10.1371/journal.pone.0302257

**Published:** 2024-04-29

**Authors:** Aleksandra Zlatkovic, Vesna Gojkovic, Jelena Dostanic, Veljko Djuric

**Affiliations:** Faculty of Legal and Business Studies, Department of Psychology, Dr Lazar Vrkatić, Novi Sad, Serbia; Novi Sad School of Business, SERBIA

## Abstract

According to biobehavioral synchronicity model, empathy—a fundamental requirement for reciprocal and prosocial behavior—is at the core of rebound from stress, an essential feature of resilience. However, there are also reports on antagonistic traits—characterized by empathic deficit—bolstering immunity to stress. In the literature there is also inconclusive evidence regarding gender-related differences in resilience. In separate female and male subsamples we analyzed the network constellation entailing resilience (assessed as rebound from stress), empathic (cognitive empathy, affective resonance, and affective dissonance) and antagonistic personality traits (Machiavellianism, grandiose- and vulnerable narcissism). For both genders, Machiavellian agency instigated by narcissistic admiration occupied the central position in the network indicating that personality’s resources for proactivity and control are essential for successful rebound. Empathy, and in particular its affective component, occupied only a peripheral position in the network. Machiavellian antagonism in men and grandiose narcissism in females bridged prosocial mechanism of resilience with antagonistic nodes of the network. In the female subsample both types of malign narcissism (rivalry and vulnerable narcissism) directly thwarted rebound. This process was not detected in the male subsample network dominated by antagonism. That is, gender-related differences were associated with the avoidance strategies rather than with the proactive strategies. Thus, resilience assessed as rebounding from stress primarily involves personality resources which modulate proactive- and prosocial- but not necessarily reciprocal behavior.

## Introduction

### Background

Numerous studies [1,2] have established resilience as one of the basic criteria for mental health and personal wellbeing. However, there is no universal agreement regarding the definition of this construct. Feldman [3] points at evolutionary importance of individual’s resilience; an asset and a strong point originating from neurobiological bases of maternal affiliation. According to Feldman, resilience first arises in the earliest synchronous mother/child dyadic relationship and upholds all future synchronous empathic social interactions. In this way, resilience is not only an indicator of personal (dys)functionality but is also among constituting factors of prosociality. However, there is also contradicting evidence indicating that while early parental deprivation stimulates development of socially aversive traits and empathic deficits [4] it also stimulates stronger resilience [5]. Thus, currently there is no unequivocal evidence on the perplexing relationships among resilience, empathy, and socially aversive traits.

In the ongoing studies on protective and risk factors of resilience the data have been analyzed in compliance with the standard latent variable model, the premise that the visible covariance among the traits is best explained as a manifestation of an underpinning latent variable. Accordingly, these studies fail to report evidence on convoluted direct and indirect relations in the constellation of personality traits predisposing to resilience; the structural relationships between resilience and its protective and risk factors. Here, we present a structural approach to resilience as an alternative and a complement to the standard latent variable procedure. This statistical methodology was selected since puzzling reality of resilience calls for an alternative analytic approach which provides information not accessible by the standard latent variable model. In the structural model presented below, prosocial traits such as empathy (implicating cooperation and reciprocity) and socially aversive traits such as Machiavellianism and narcissism (implicating self-centeredness and antagonism) are assumed to directly affect each other.

### Resilience

Resilience entails not only a capacity to deal with stress through use of adequate coping mechanisms but also an ability to rebound from negative experience [6]. Individual differences in resilience are shaped by one’s personality, affective and cognitive capacities, and the overall quality of interpersonal relationships; while there is inconclusive evidence regarding gender-related differences in resilience [7]. Capacity for cognitive processing of stressful events and positive affect [8] are positively related to resilience. High level of Neuroticism impedes resilience, in as much as high levels of other basic personality traits—in particular Conscientiousness (as a control system) and Extraversion (as activity and positive affect)—are positively related to high resilience [9]. Typological studies confirm that personal capacities for activation and control (Extraversion and Conscientiousness) are protective while affectivity (high Emotionality, Neuroticism, and Introversion) is a risk factor of resilience [10]. At the same time, affectivity is an essential component of empathy; and thus indispensible for strengthening of growth-fostering relationships.

### Resilience and empathy

Empathy is a fundamental requirement for reciprocal and prosocial behavior as it involves both the cognitive skill for recognizing emotions and the ability for harmonizing one’s emotional state with affective states of other people. Though it is usually held that strengthening of resilience also bolsters proclivity for empathy [3], the relationship between the two constructs may not be so straightforward, after all. Thus, it seems that positive association between resilience and empathy holds only for women but not for men [11]. Some studies report that empathy is a typical feature of women [12], while other studies report that heightened resilience is predominantly found in men [7]. Similarly, some studies indicate that early parental deprivation hinders empathy and intensifies vulnerability in children, while other studies suggest that childhood maltreatment predicts immunity, or resilience to stress [13], an undisputable component of psychopathy and other antagonistic or callous–unemotional traits [14], like Machiavellianism and narcissism. So, the link between resilience and empathy is more complex than what is usually supposed: high resilience is not necessarily associated with high empathy, and vice versa.

### Empathic deficit as a criterion for maladaptation

As opposed to resilience, empathic deficit is not only a criterion of personal dysfunctionality, but is also a criterion of social maladaptation. Vachon and Lynam [15] point out that—besides its cognitive component and affective resonance—empathy involves affective dissonance, as well. Affective resonance involves a reciprocal emotional response to an emotional stimulus (e.g., empathy, sympathy, compassion). Affective dissonance (e.g., sadism, scorn, schadenfreude) outperforms low affective resonance as a predictor of pathological personality traits. Affective dissonance is the underpinning of antagonism [16], the joint fabric of psychopathy, narcissism and Machiavellianism. Expression and the joint occurrence of these personality traits are primarily reported in men [17]. Interestingly, callousness may be positively associated with some positive outcomes in response to environmental demands such as emotional resilience and high self-esteem [5]. Absence of emotionality and low levels of anxiety [18] become manifest as cold-blooded and cool-minded behavior in stressful situations and greatly enhance adaptation under nerve-racking circumstances [19]. Nonexistence of affective response facilitates rational evaluation or “cognitive reformulation” that is quite helpful when examining the optimal coping strategy [20]. Besides, high emotional sensitivity leads to vulnerability and is an undeniable risk factor of resilience. HEXACO model of personality structure posits that Emotionality and Resilience are the opposite poles of the basic personality trait [21].

Additionally, resilience is characterized by outstanding regulation of strong emotional responses, staying focused in the face of hardships, and flexible adaptation to environmental changes [22]; notwithstanding the fact that these qualities are often found in people marked with pronounced antagonistic personality traits such as Machiavellianism and narcissism.

### Narcissism

Narcissism is a heterogeneous construct behaviorally presented through its overt (grandiose) and covert (vulnerable) manifestations. Grandiose narcissism is characterized by self- aggrandizement, vanity, exhibitionism, manipulation, and interpersonal domination; vulnerable narcissism by negative affect, distrust, selfishness, attention seeking, and entitlement [23]. Grandiose narcissism negatively correlates with Agreeableness and Neuroticism; and positively with the narcissistic personality disorder [23], Extraversion, risk taking, status seeking, and even with some socially desirable behaviors, such as empathy [24]. According to Back and his colleagues [25], these divergent outcomes (like positive correlation between grandiose narcissism and empathy) are due to the dual nature of grandiose narcissism. Admiration—involving assertiveness, self-confidence, dominance, and allure—is the interpersonal component of grandiose narcissism, quite conducive for gaining praise and recognition of others and for further nurturing of the already bloated self-portrayal. Rivalry—the intrapersonal component of grandiose narcissism and its antagonistic core—is activated by any threat to this self-absorption. In addition to its obvious self-centered and antagonistic dimension, grandiose narcissism is characterized by its incessant need for approval and admiration. In order to satisfy this urge, narcissists often engage in socially highly esteemed actions and project signs of care and compassion. Narcissists use their ability to correctly recognize emotions of other people in order to foster their manipulative interpersonal strategies [16]. Admiration is positively associated with Extroversion and socially desirable behaviors [26] while rivalry, accompanied by affective dissonance, is at the very heart of antagonism [16]. Admiration is considered to be protective while rivalry is reported to exacerbate psychological distress [27].

Quite distinctive from the grandiose narcissism, vulnerable narcissism is foremost characterized by feelings of helplessness and being victimized [23]. Interpersonal strategy of vulnerable narcissism is best understood as a persistent manipulation with one’s susceptibility to pain in order to satisfy authentic egotistic urges [28]. Vulnerable narcissism correlates with avoidant and dependent personality disorders and grandiose narcissism [23]. Grandiose narcissism is more prevalent in men, while vulnerable narcissism is either gender-neutral or more prevalent in women [29]. Antagonism (low Agreeableness) constitutes the common core of both the grandiose and the vulnerable narcissism. Grandiose narcissism is visible by high Extraversion and low Neuroticism while vulnerable narcissism is evident by low Extraversion and high Neuroticism [30]. Only the vulnerable—but not the grandiose—narcissism is negatively correlated with cognitive and affective empathy and with distinct dimensions of resilience [31]. Grandiose narcissism is negatively and vulnerable narcissism is positively associated with perceived stress [32]. Furthermore, only the grandiose narcissism—but not the vulnerable narcissism—constitutes the dark triad, together with psychopathy and Machiavellianism.

### Machiavellianism

Machiavellianism is a personal trait fixed in the negative view of human nature and destructive interpersonal tactics [33]. It is a significant predictor of intimidation, betrayal, duplicity, deceit [34], and alexithymia [35]. While Machiavellianism is closely related to psychopathy [36], jointly forging the core of evil, it is not devoid of some desirable components such as strategic planning, good impulse control, and insensitivity to provocation [34]. Taken together, these findings suggest its complex configuration and the need for multidimensional measurement of Machiavellianism. According to Collison and colleagues [37], Machiavellianism is constituted of antagonism (its core component), agency, and planfulness; factors saturated by Machiavellian goal-oriented strategies. Antagonism is composed of callousness, cynicism, manipulation, and egocentrism. Agency consists of assertiveness, feelings of competence, emotional invulnerability, and self-confidence resulting from low Conscientiousness. Planfulness is based on premeditation and orderliness. Predictive association between Machiavellianism and resilience remains somewhat controversial. While grandiose narcissism is recognized as a positive predictor of resilience [31], there are reports on both negative and moderating and positive associations between Machiavellianism and resilience [38]. Only Machiavellianism, but not grandiose narcissism, is associated with negative perception of stressful situations [39]. It is quite possible that this incongruence may be a consequence of unalike conceptualizations of variables among the studies, but also a consequence of the standard latent variable approach which is insensitive to direct pairwise interactions of study variables and their spatial arrangements. To the best of our knowledge, we still do not have any structural evidence about resilience as defined by its bivariate interactions with empathy, Machiavellianism, and with the grandiose and the vulnerable narcissism.

### The current study

Here we tried to obtain answers to three separate yet interconnected questions.

First, we investigated a convoluted relationship between resilience and empathy. According to Feldman [3] sociality, incorporating reciprocity and empathy, is among the three pillars of resilience. However, there is ample evidence that empathy may impede resilience while absence of empathy may build it up [13]. Second, lack of empathy is a common feature of antagonistic personality traits [15] such as Machiavellianism and grandiose and vulnerable narcissism. Although our knowledge about predictive value of these antagonistic traits is still inconclusive, it is obvious that each one of them has a distinctive predictive relationship with resilience. Third, studies indicate gender-related differences in resilience: higher empathy and lower resilience in women accompanied by lower empathy and higher resilience and antagonism in men. Given that all these studies were analyzed under the standard latent variable model it may be that structural analysis could provide pertinent gender-related information about spatial constellation of study variables not accessible by other means.

The primary objective of this study was to ascertain structure of resilience in a network consisting of empathy (implying reciprocal and prosocial behavior), Machiavellianism, and grandiose and vulnerable narcissism (indicating self-centered and socially aversive behavior); independently for each gender. In specific, we tested predictions of the biobehavioral synchronicity model against predictions of the view emphasizing a bolstering effect of antagonism on rebound.

### Hypotheses

H1: In compliance with the corollary of the paradigm proposed by Feldman [3] that resilience is strengthened through empathic and cooperative social interactions, we assume that in both genders the structure of resilience will be defined by direct positive connections with the cluster of traits involving prosocial reciprocity. Besides the obvious assembly of cognitive empathy and affective resonance this cluster entails socially desirable manifestations of narcissism and Machiavellianism: admiration, agency and planfulness. Simultaneously, resilience will be negatively and indirectly connected with antagonistic traits: vulnerable narcissism, rivalry, affective dissonance, and Machiavellian antagonism.
H2: Earlier studies on resilience [10], consistently confirm primary protective role of personality resources for control and activity. In this study, Machiavellian agency—when its shared variance with the core Machiavellian dimension of antagonism is partailed out—connotes the very potentials necessary for activation of these resources. Therefore, we predict that, irrespective of gender, agency will occupy the central position in the network. That is, agency will have the strongest direct positive link with resilience and at the same time will bridge the cluster of prosocial with the cluster of socially aversive and self-centered personality traits.
H3: Vulnerable narcissism will be the dominant direct inhibitor of resilience in both genders due to its saturation with negative affect and with interpersonal antagonism entrenched in the subjective feeling of being a victim. Namely, narcissistic hypersensitivity leads to conflict with others and is completely unalike proactive and controlled behavior that is obligatory for successful rebound [32].
H4: With all this in mind, we assume only quantitative gender-related differences with respect to structure of resilience. In particular, the resilience network in men will be characterized by stronger direct contribution of antagonism [17].

## Method

### Participants

This on-line study enabled by the Google forms service involved a female and a male subsample. The questionnaires were posted online on different social media. Incomplete responses and responses of participants younger than 18 were removed from all analyses. Female subsample consisted 677 females (M_age_ = 31.68; SD_age_ = 10.57) while male subsample consisted of 133 males (M_age_ = 28.14; SD_age_ = 9.22). The data were collected from July 1, 2022 to August 31, 2022. All participants provided a written informed consent for their voluntary participation in the study. The study was approved by the Ethical Committee of our institution. The study met all ethical requirements in agreement with the Declaration of Helsinki and the legal requirements of the Republic of Serbia. The sample data are available at OSF.

### Psychometric scales

Resilience was assessed by the Serbian translation of the Brief Resilience Scale—BRS [40]. The scale includes six 5-point Likert items. The total BRS score indicates the ability to bounce back or recover from stress. Higher BRS score implies higher resilience. At the time of our study, there was no standardized Serbian version of the BRS. Therefore, the validity of the single-factor solution was assessed using confirmatory factor analysis (CFA) conducted in R (version 4.3.2) with the *lavaan* package and a maximum likelihood estimator [41]. As the initial one-dimensional model yielded substandard fit, an alternative model allowing for two error covariances among the items was introduced. This modification resulted in a satisfactory model fit [42,43]: χ2/df ≤ 5; CFI = .99; RMSEA = .07; SRMR = .02. Detailed results of the CFA can be found on OSF.

Empathy was assessed by the Serbian translation of the Affective and Cognitive Measure of Empathy—ACME [15] consisting of 36 self-report items. ACME entails 3 subscales: cognitive empathy, affective resonance, and affective dissonance. The items were administered using a 5-point Likert scale. For calculation of the total ACME score affective dissonance scores have been reversed so that the total ACME score represents the overall measure of empathy, with higher ACME scores reflecting higher empathy.

Grandiose narcissism was assessed by the Serbian translation of Narcissistic Admiration and Rivalry Questionnaire—NARQ [25] consisting of eighteen 5**-**point Likert**-**type items that measure two dimensions of narcissism: the socially desirable narcissistic admiration and rivalry, the antagonistic essence of narcissism. The original 7-point Likert type rating was replaced with a 5-point rating scale. This was done in order to secure equidistance as an essential feature of interval measurement since 1–5 grading is uniformly used in the Serbian school system and therefore was more familiar to our respondents. Higher score on each NARQ dimension reflects more pronounced presence of a given trait.

Vulnerable narcissism was assessed by the Serbian translation of the Narcissistic Vulnerability Scale—NVS [44]. In the present study the original form of the NVS scale (11 adjective-based items) was converted to a 5-point Likert scale. The total NVS score indicated the extent of vulnerable narcissism. At the time of our study, a standardized Serbian version of the NVS was unavailable. Consequently, we assessed the validity of the single-factor solution through CFA conducted in R (version 4.3.2) using the *lavaan* package and a maximum likelihood estimator [41]. The initial one-dimensional model, which lacked error covariances, resulted in an unsatisfactory fit. Subsequently, an alternative model was proposed, incorporating seven paths of covariance errors among the items, leading to a satisfactory fit [42,43]: χ2/df ≤ 5; CFI = .94; RMSEA = .07; SRMS = .05. Detailed results of the CFA are available on OSF.

Machiavellianism was assessed by the Serbian translation of the 52-item form of the Five Factor Machiavellianism Inventory*—*FFMI [37]. FFMI was created to assess three dimensions of Machiavellianism: antagonism (selfishness, immodesty, manipulativeness, callousness, and cynicism), agency (activation, assertiveness, competence, invulnerability, and self-confidence) and planfulness (deliberation and order). The total FFMI score indicated the extent of Machiavellianism.

Please note that there are official adaptations of the used NARQ [https://osf.io/vq7gu/], FFMI [20,45], and ACME [46] instruments, but in this study we used different adaptation because we were not aware of official adaptations in the period of data collection.

### Data analysis

In the first stage, by use of SPSS software version 25, descriptive statistics, Cronbach alpha, and Pearson product moment correlations were analyzed separately for both (male and female) subsamples. In the second stage, network analysis was used to directly assess bivariate partial correlations among the individual study variables and the ensuing topology of the data set.

### Why network analysis

Central assumption of the latent variable model postulates that underlying constructs (abilities, traits) are not directly observable but can be inferred from observed/measurable/manifest variables. Relationships between the latent and the observed variables are specified by the covariance matrix of the observed variables. The main objective of the latent variable model is to identify strength of the relationships between latent and manifest variables and to identify the yet unexplained variance of observed variables. Originated in the seminal Hotelling’s work on the principal component analysis in the 1930’s, over the decades latent variable model has expanded to various specific forms including exploratory and confirmatory factor analysis and structural equation modeling. Thus, analytic potentials of the latent variable model have been validated in countless psychological studies. Along the way it has earned the prefix “standard” and is today universally known as the standard latent variable model. In contrast to the standard latent variable approach, the network model utilized in this study rests on the assumption that complex relationships among study variables are best explained by pairwise partial correlations defining a network structure [47].

Structural analysis is based on the premise that study variables directly affect each other by establishing a unique structure of variables’ network [47]. Thus, structural analysis concentrates on pairwise relationships between observed variables and the topology of the resulting network of their bivariate associations. By use of indicators of centrality (how essential is a trait for the overall network topology) and redundancy (a degree to which a trait is replaceable with other traits from the network), structural analysis provides insight and visualization of bivariate relationships among the variables. The network analysis on both subsamples was performed by JASP (version 0.16.3) utilizing regularized EBICglasso estimation method, which features high specificity while not estimating those edges that are not in true network, and demonstrates varying sensitivity [48]. The network consisted of nodes representing observed variables and edges representing regularized partial correlations between two variables after controlling for all other variables. Absence of a direct edge between the two nodes means that they are not related to each other after controlling for the contribution of other nodes in the network. Centrality of variables was assessed via indices of strength, closeness, and betweenness, accompanied by centralized Zhang clustering coefficient indicating node redundancy [49]. Nonparametric bootstrap on 1000 samples was used for assessing edge-weight accuracy and case-dropping bootstrap was used for assessing reliability of centrality indices (OSF).

## Results

Descriptive statistics, Pearson bivariate correlations and reliabilities of study variables for each gender are presented in Table 1. Due to a high probability of Type I error bivariate correlations are primarily presented for descriptive purposes.

**Table 1:** Inter-correlations, means, standard deviations, and internal consistency for scores on the BRS, ACME, NARQ, NVS and FFMI scales on female and male subsamples.

	*Mean*	*SD*	1	2	3	4	5	6	7	8	9	10	11	12	13
1 GN	2.29	.41	.*75*	.83**	.78**	.21*	.48**	.49**	.31**	.07	-.31**	.09	-.28**	.50**	-.07
2 GN_Adm	2.88	.63	.88**	.*79*	.26**	-.02	.49**	.30**	.48**	.08	-.08	.20*	-.11	.28**	-.01
3 GN_Riv	1.71	.41	.68**	.23**	.*60*	.38**	.28**	.49**	-.01	.03	-.43**	-.07	-.36**	.54**	-.10
4 Vuln_narc	2.36	.64	.11**	-.14**	.43**	.*80*	-.22*	.27**	-.51**	-.14	-.22**	-.04	-.15	.30**	-.17*
5 M	2.92	.36	.45**	.50**	.15**	-.31**	.*81*	.68**	.82**	.39**	-.22**	.24**	-.44**	.30**	.20**
6 M_Ant	2.24	.49	.33**	.16**	.42**	.24**	.59**	.*78*	.22**	-.04	-.58**	-.01	-.70**	.61**	-.03
7 M_Act	3.31	.56	.37**	.54**	-.09*	-.58**	.82**	.08*	.*85*	.23**	-.04	.25**	-.15	.02	.29**
8 M_Plan	3.46	.58	.04	.07	-.03	-.07	.39**	-.02	.21**	.*50*	.30**	.31**	.17	-.19*	.13
9 E	4.40	.36	-.09*	.10*	-.32**	-.23**	-.05	-.43**	.19**	.17**	.*85*	.62**	.83**	-.79**	.08
10 Cog_Emp	4.00	.68	.10**	.21**	-.12**	-.14**	.19**	-.06	.26**	.11**	.75**	.*89*	.22**	-.15	.16
11 Aff_Res	4.50	.47	-.12**	.01	-.26**	-.14**	-.22**	-.53**	-.03	.15**	.77**	.26**	.*77*	-.65**	-.04
12 Aff_Dis	1.30	.35	.30**	.13**	.40**	.25**	.21**	.49**	-.04	-.09*	-.55**	-.01	-.50**	.*71*	-.05
13 Resilience	3.35	.93	.10*	.25**	-.19**	-.47**	.38**	-.09*	.57**	.06	.20**	.21**	.07	-.11**	.*84*

^*^ *p* < .05

** *p* < .01.

*Note*. Zero–order correlations are presented: below the diagonal for female subsample and above the diagonal for male subsample; In diagonal, in italic, the Cronbach’s alpha coefficients.

GN = grandiose narcissism; GN_Adm = admiration; GN_Riv = rivalry; Vuln_narc = vulnerable narcissism; M = Machiavellianism; M_Ant = antagonism; M_Act = agency; M_Plan = planfulness; E = empathy; Cog_Emp = cognitive empathy; Aff_Res = affective resonance; Aff_Dis = affective dissonance.

There were 52 statistically significant correlations in the female subsample. Resilience was significantly correlated with all study variables except planfulness and affective resonance. Interestingly, resilience was more closely associated with the total Machiavellianism score (sharing 14% of the common variance) than with the total empathy score (only 4% of the common variance). Resilience’s strongest association was with agency (30% of the common variance), followed by the negative association with vulnerable narcissism (22% of the common variance). Resilience was also positively associated with admiration and cognitive empathy, sharing approximately 6% of the common variance with both. Resilience also negatively correlated with rivalry (4% of the shared variance), affective dissonance (1% of the shared variance) and antagonism (less than 1% of the shared variance). Markedly, vulnerable narcissism was negatively associated with agency (34% of the shared variance); antagonism was negatively associated with affective resonance (28% of the shared variance); while antagonism was positively associated with affective dissonance (having approximately 24% of the shared variance).

There were 48 statistically significant bivariate correlations in the male subsample. Resilience was significantly–albeit weakly–correlated only with three study variables. The strongest association was with Machiavellian agency (9% of the shared variance), followed by the total score on Machiavellianism (4% of the shared variance) and the negative association with vulnerable narcissism (3% of the shared variance). The strongest negative correlation was between antagonism and affective resonance (sharing 49% of variance) followed by its positive correlation with affective dissonance (36% of the common variance) and its negative association with the total empathy score (sharing 25% of variance). Vulnerable narcissism was negatively correlated with agency (sharing 25% of the common variance), while rivalry positively correlated with affective dissonance and negatively with affective resonance (sharing 25% of variance with each). Grandiose narcissism was positively associated with Machiavellianism and antagonism (sharing 23% of variance with each), and with agency (sharing 9% of the common variance). Admiration was positively correlated with vulnerable narcissism and agency (sharing approximately 24% of variance with each). Positive correlation between rivalry and antagonism accounted for 25% of their common variance. In comparison with the female subsample, male subsample was represented by strong positive correlations among the antagonistic personality traits and their negative associations with empathy and particularly with affective resonance.

### Network analysis of the female subsample

**Fig 1:**
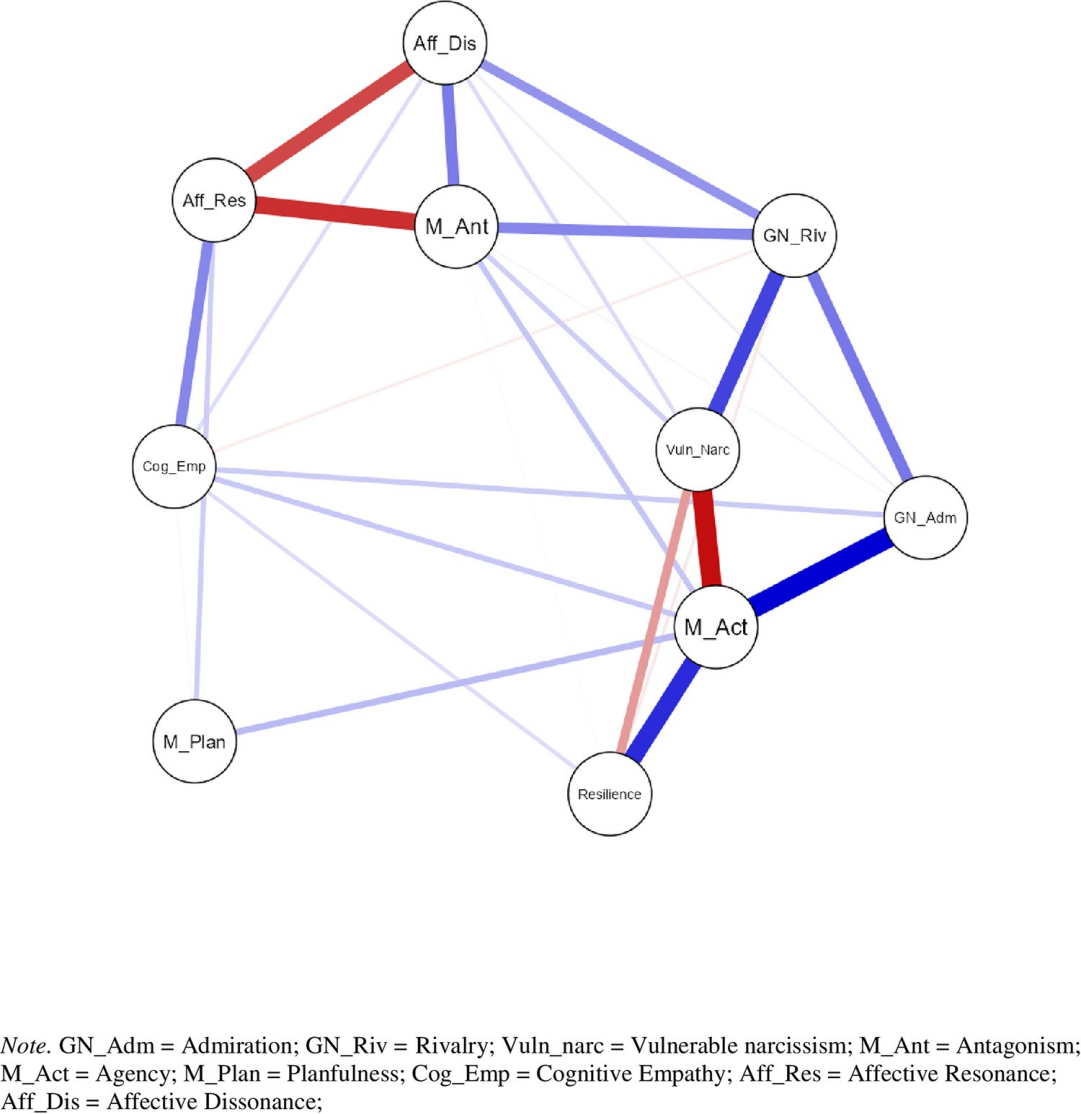
Estimated network structure of BRS, ACME, NARQ, NVS and FFMI dimensions of the female subsample.

By use of the EBICglasso methodology, the topology of study variables was represented as 10 nodes connected by 27 (out of 45 possible) non-zero edges depicting the strength and direction of pairwise regularized partial correlations. The network topology is illustrated in Fig 1. Positive associations are colored by blue while negative associations are colored by red lines. The thickness of an edge corresponds with the strength of the association.

**Fig 2:**
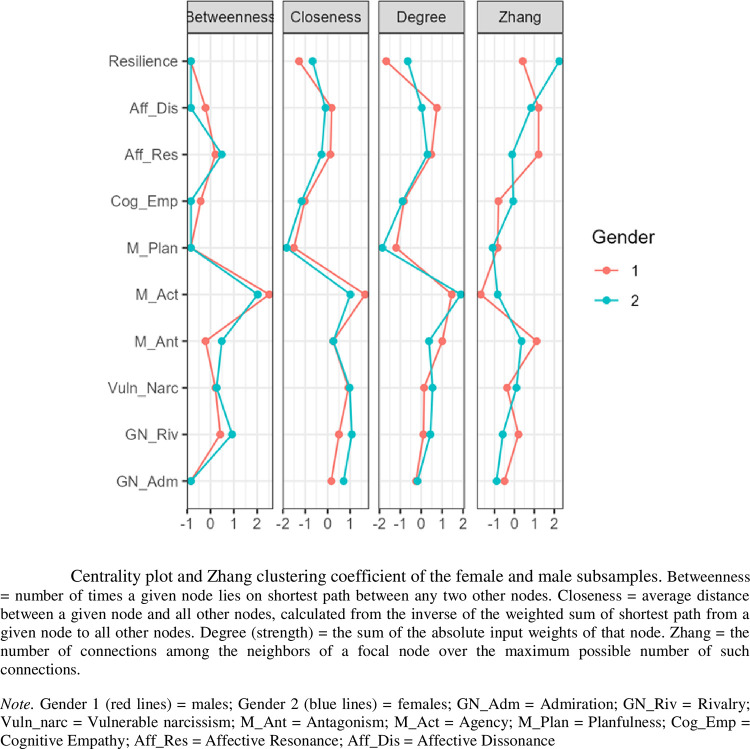
Centrality plot and Zhang clustering coefficient of the female and male subsamples.

The highest positive regularized partial correlations (Table 2) were depicted as links between agency and admiration (sharing 17% of the unique variance), agency and resilience (sharing 12% of the unique variance), and rivalry and vulnerable narcissism (sharing 9% of the unique variance). Likewise, the highest negative regularized partial correlations were depicted as links between agency and vulnerable narcissism (sharing 15% of the unique variance), and agency and affective resonance (sharing 11% of the unique variance). The most central place in the network was occupied by Machiavellian agency **(**as displayed by all three centrality indicators; Fig 2)**—**followed by vulnerable narcissism and rivalry. Vulnerable narcissism was more central than rivalry with respect to absolute edge weights connected to each node (strength); but rivalry was more central than vulnerable narcissism with respect to how often one node was in the shortest path between other nodes (betweenness), and the inverse of the sum of distances from one node to all other nodes in the network (closeness).

**Table 2:** EBICglasso partial correlations from network analysis for BRS, NARQ, NVS, FFMI, and ACME dimensions on the female and the male subsample.

	1	2	3	4	5	6	7	8	9	10
1 Resilience		0	0	0	0	.16	0	.03	0	0
2 GN_Adm	0		.13	0	.05	.32	0	.07	0	.08
3 GN_Riv	-.04	.22		.20	.18	0	0	0	0	.25
4 Vuln_Narc	-.16	0	.30		.09	-.41	0	0	0	.07
5 M_Ant	-.01	.01	.20	.08		.16	0	0	-.43	.18
6 M_Act	.34	.41	0	-.39	.09		.08	.08	0	0
7 M_Plan	0	0	0	0	0	.11		.20	.01	-.07
8 Cog_Emp	.06	.08	-.03	0	0	.09	.01		.10	0
9 Aff_Res	0	0	0	0	-.34	0	.08	.20		-.34
10 Aff_Dis	0	.3	.17	.06	.21	0	0	.06	-.30	

*Note*. EBICglasso partial correlations from the female subsample are given in the lower triangle of the matrix; EBICglasso partial correlations from the male subsample are given in the upper triangle of the matrix; GN_Adm = admiration; GN_Riv = rivalry; Vuln_narc = vulnerable narcissism; M_Ant = antagonism; M_Act = agency; M_Plan = planfulness; Cog_Emp = cognitive empathy; Aff_Res = affective resonance; Aff_Dis = affective dissonance.

A strong positive edge connected rivalry and vulnerable narcissism, and both nodes (though with widely different intensities) were negatively connected to resilience. Regularized partial correlations (edges) of resilience with vulnerable narcissism, rivalry, and cognitive empathy were unimpressive, with edges never accounting for more than 2% of the unique shared variance save for its association with agency. Partial pairwise correlations between resilience and all other study variables were limited to zero by the regularized EBICglasso estimation method as they were eclipsed by agency, the most central node of the network.

In addition to its direct yet weak link with resilience, rivalry was significantly positively connected to vulnerable narcissism and somewhat less intense with antagonism and affective dissonance. Rivalry was connected with activity and resilience via admiration, its external component. Hence, rivalry held an important spot in the network by bridging the two prevailing clusters of nodes. The one centered on Machiavellian antagonism (containing a negative edge with affective resonance and a positive edge with affective dissonance) and the other centered on Machiavellian agency (entailing a negative edge with vulnerable narcissism and positive edges with admiration and resilience). Vulnerable narcissism was directly positively connected with antagonism and affective dissonance, though to a lesser extent than with resilience and agency. Due to absence of any direct edge, vulnerable narcissism has lost much of its shared variance with admiration relative to the common variance underpinning their zero-order correlation. In the network, vulnerable narcissism was only indirectly connected to admiration via agency and rivalry.

Peripheral position of planfulness and cognitive empathy was evidenced by their lowest centrality indices. However, according to their Zhang clustering coefficients they should not be labeled as redundant as their exclusion would profoundly affect network’s topology. Resilience was the most redundant node in the network. As resilience has been operationalized as a behavioral outcome, by definition occupying the lowest level in the hierarchy of personality traits/study variables, a reliable interpretation of its redundancy is devoid of any psychological meaning. As the principle objective of network analysis was to ascertain positioning of antagonistic and prosocial traits relative to resilience, this finding will not be a subject of the ensuing discussion. Save for resilience, affective dissonance was the most redundant node of the network. Strong positive coupling between affective dissonance and antagonism, and their common positive link with rivalry and a common negative link with affective resonance shaped the antagonistic cluster of the network with no direct links with resilience. Finally, cognitive empathy was directly weakly connected to resilience, agency and admiration with each accounting for approximately only 1% of the unique shared variance.

### Network analysis of the male subsample

**Fig 3:**
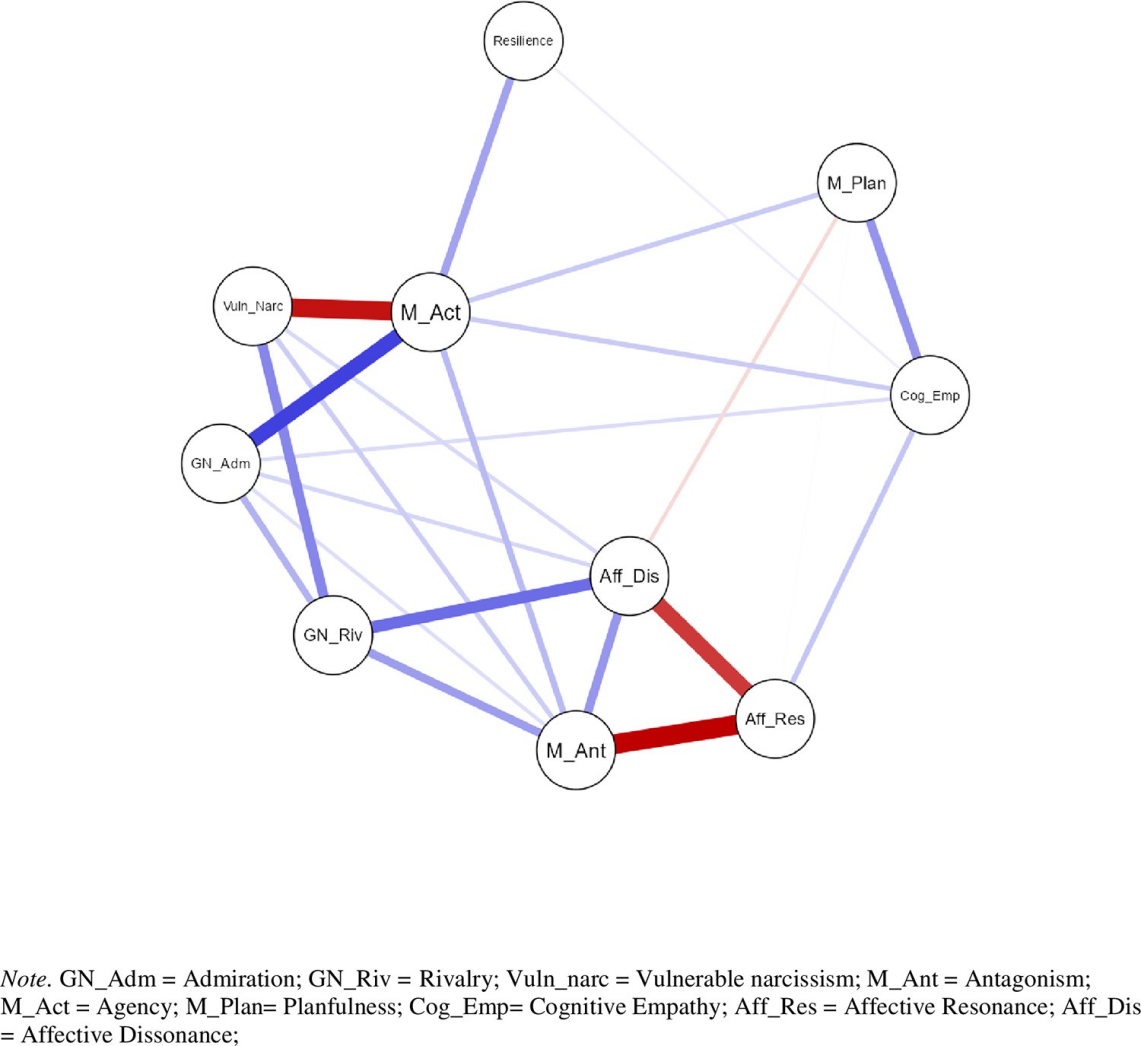
Estimated network structure of BRS, NARQ, NVS, FFMI and ACME dimensions of the male subsample.

A 10-node network yielded 23 out of 45 possible non-zero edges. The network topology is illustrated in Fig 3. The strongest edge of the network was the negative edge connecting affective resonance and Machiavellian antagonism, accounting for 19% of the unique shared variance (Table 2). Strength-wise it was followed by the negative pairwise partial correlation between vulnerable narcissism and agency (17% of the unique shared variance) and the negative link between affective resonance and affective dissonance (responsible for 12% of the unique shared variance). The strongest positive edge connected admiration and agency, with two nodes sharing 11% of the unique variance. Resilience, quite isolated in the network topology, was directly connected only with agency (sharing 3% of the unique variance) and cognitive empathy (sharing 1% of the unique variance). Positive regularized partial correlation between resilience and agency was significantly lower in the male subsample (female *r*_*parc*_ = .34, male *r*_*parc*_ = .16; *z*-score = 2.01, *p* = .04). Contradictory to their zero-order correlation (Table 1) the network link between resilience and vulnerable narcissism was mediated through agency, as agency has taken over substantial proportion of variance of vulnerable narcissism (the second strongest negative edge of the network). Low positive regularized partial correlations were identified between affective dissonance and rivalry (6% of the unique common variance), vulnerable narcissism and rivalry (4% of the unique common variance), planfulness and cognitive empathy (4% of the unique common variance), antagonism and rivalry (3% of the unique common variance), antagonism and agency (2% of the unique common variance), admiration and rivalry (2% of the unique common variance). Regularized partial correlations between agency and cognitive empathy, admiration and affective dissonance, vulnerable narcissism and affective dissonance, and admiration with agency and cognitive empathy, separately accounted for less than 1% of the unique shared variance.

According to all 3 indices of centrality, Machiavellian agency occupied the most central position in the network, as was the case with the female subsample (Fig 2). Degree-wise, antagonism was the second strongest node of the network owing to its direct connections with nodes from all three measuring domains (Machiavellian, narcissistic, and empathic). With respect to the closeness criterion (the sum of the absolute input weights) vulnerable narcissism was the second strongest node of the network. Resilience occupied a peripheral position due to low values on all three centrality indices, but was not redundant. According to the Zhang clustering coefficient, affective dissonance was the most redundant node of the network, followed by affective resonance and Machiavellian antagonism. The links between antagonism and both affective components of empathy were among the strongest edges in the network. Consequently, omission of any those nodes would not substantially affect the overall topology. Predominance of edges connecting antagonistic nodes in the male network largely overshadows edges between resilience (a personality trait of a lower order) and any other nodes.

## Discussion

Overall, our data suggest that in both genders resilience was primarily facilitated by Machiavellian agency and inhibited by vulnerable narcissism. This implies a secondary role of empathic mechanisms since they are involved only as a cognitive ability for knowing how other people think and feel. Thus, rebounding from stress involves personality resources which modulate proactive—and prosocial—but not necessarily reciprocal behavior. In the female subsample antagonistic dimensions of narcissism (rivalry and vulnerable narcissism) directly inhibited the rebound, whereas in men there was no direct inhibition of resilience. Machiavellian antagonism in men and grandiose narcissism in females bridged prosocial mechanism of resilience with antagonistic nodes of the network. Structural gender-related differences in resilience were determined by rather weak yet statistically significant regularized partial correlations. As such, they point at the essential difference between rebound mechanisms in two genders.

This study further documents the central protective role of Machiavellianism agency. Finally, the study has demonstrated that both grandiose and vulnerable narcissism significantly affect the rebound mechanism, yet in a gender specific manner.

### Resilience structure in females: The resilience facilitation and the resilience inhibition axes

The network analysis on the female subsample set apart the two axes: the facilitating axis consisting of agency—admiration and agency–resilience edges, and the inhibitory axis consisting of agency–vulnerable narcissism and the rivalry–vulnerable narcissism edges. The first axis was dominated by Machiavellian proactivity and the latter by the inhibitory action of both the grandiose and the vulnerable narcissism, in accordance with our H1.

#### The central role of Machiavellian agency

Successful rebound calls for resources containing activity and control, which are equally contained in Machiavellian agency [37]. Proactivity, good impulse control, goal directed behavior devoid of emotional interference, and focusing on one’s own competences are all predictive of successful recovery form stress. This finding is in line with previous studies on resilience including both genders [31], dissimilar operationalization of resilience [50]; and categorical data analysis [10]. In our female subsample, capacity for proactivity was strongly instigated by agentic narcissism, admiration. This was the strongest edge in the network powered by strategies aimed at avoidance of interpersonal conflicts, and consequently the main driving force of the rebound, thus retiring affect to only a peripheral contribution. Indirectly, our data support the notion that empathic affects may thwart resilience since it intensifies sensitivity and leads to empathic distress [51].

Narcissistic vulnerability and (to a lesser degree) narcissistic rivalry as the main proponent of antagonism in the network, are risk factors directly imperiling the rebound by suppression of proactive behavior. Proclivity for conflict and fragility do not contribute to a goal oriented behavior. Cognitive empathy enhances resilience directly, but also within a frame of proactive executive strategies utilizing the ability for being aware of hearts and minds others in order to make a rational decision. This mechanism of rebound is prosocial, but not necessarily reciprocal thus only partially confirming our H1.

#### Grandiose narcissism as the main bridge of the network

In women, both components of grandiose narcissism are involved in resilience mechanism albeit in an opposing fashion. The strong direct edge connecting admiration and agency depicts the route by which admiration additionally reinforces proactivity of Machiavellian agency, consequently enhancing resilience. Absence of the direct edge between admiration and resilience, in spite of the significant Pearson’s product moment correlation between the two variables, accompanied by the strong edge linking admiration and agency indicates that Machiavellian agency has taken over the greatest share of the common variance. Besides testifying about the shared agentic nature of the two essentially aversive traits, this network configuration accentuates the prominent role of narcissistic admiration in the dynamics of rebound [27].

Rivalry was the chief representative of antagonism in the network. It is the only node directly positively connected to affective dissonance, Machiavellian antagonism, and vulnerable narcissism and concurrently negatively connected to resilience. Quite in line with the proposed theoretical model [25], rivalry is connected to other nodes of the network via admiration, its conceptual counterpart. In accordance with previous findings [27], rivalry is a direct suppressor of resilience. Likewise, rivalry facilitates vulnerable narcissism hence indirectly inhibiting successful rebound. Therefore, the data from the female subsample only partially confirm our H2 since agency was the central node of the network but grandiose narcissism was the bridge connecting prosocial and antagonistic nodes.

#### Vulnerable narcissism as the main direct inhibitor of resilience

The network analysis confirmed our H3 since vulnerable narcissism was identified as the main negative direct predictor of resilience. This finding is in agreement with reports on vulnerable narcissism’s antagonistic nature and its negative impact on resilience [31]. Vulnerable narcissism, the second strongest node of the network, is primarily defined by antagonistic narcissism, dissonant affect, and the absence of agency; the traits characterized as the ‘core of evil’ [16]. A twofold inhibitory effect of vulnerable narcissism was accomplished directly through unmediated suppression of resilience, but also indirectly via restraint of agency. The profound feeling of vulnerability is a potent deterrent of proactive adaptive behavior. For that reason, vulnerable narcissism is the main suppressor of rebound in females and accordingly the leading risk factor of resilience.

#### Resilience structure in males: The resilience and the antagonism axes

Structure of resilience in the male subsample was also composed of the two interconnected axes, in accordance to our H1. The first axis consisted of the negative edge between agency and vulnerable narcissism, the positive edge between admiration and agency, and the two rather weak positive edges connecting resilience with agency and cognitive empathy. The second axis of the resilience network consisted of tightly interconnected antagonistic nodes (strong negative edge between Machiavellian antagonism and affective resonance, accompanied by rivalry’s positive edges with vulnerable narcissism and affective dissonance), indirectly affecting the rebound.

#### The central role of Machiavellian agency

In this constellation agency was the central mechanism of rebound, as was the case in the female subsample, thus supporting our H2. As in the female subsample, proactivity was the main device of rebound, very much in compliance with previous studies [50]. In both genders, the basic mechanism of rebound presented as a coupling of agency and admiration, accompanied by a slight involvement of cognitive empathy and by an inhibitory action of both forms of narcissism; partially in accordance with our H1.

However, this mechanism is unevenly instigated by gender-related differences in predominant personality traits, and by gender-related differences in inhibition of resilience. In this sense, gender-related differences in resilience are not only of quantitative but are also of qualitative nature, thus contradicting our H4. Quantitative gender-related differences address much stronger expression of antagonism and weaker edges among the resilience promoting nodes in males. Conversely, gender-related qualitative dissimilarities in structure of the resilience mechanism are evidenced as differences in strength of edges connecting antagonistic nodes and interactions of these nodes with agency, the main forbearer of resilience.

#### Antagonistic traits dominate the whole network

Antagonism—the core component of Machiavellianism—is not only more pronounced but was also differently positioned in the men’s network. In men, Machiavellian antagonism—characterized by absence of resonant affective response and by presence of dissonant affect—was a bridge connecting the dominant antagonistic axis with the resilience axis. Therefore, it could be argued that in men there are no empathic and/or moral constrains about strategies leading to successful rebound. This is consistent with previous reports [17] on Machiavellianism and particularly Machiavellian antagonism as a dominant male trait.

Strong expression of antagonistic traits dominates the entire structure of the male network: even when Machiavellian antagonism occupied a somewhat peripheral position due to its strong negative link with affective empathy, rivalry and vulnerable narcissism held their strong links with agency. Secondly, the edge connecting Machiavellian agency and resilience was significantly weaker than in the female network. Thirdly, resilience was directly linked to only two other nodes in the male network. Finally, both affective components of empathy are deemed redundant according to the Zhang criterion. Their omission would not critically affect topology of the network. Peripheral position of both affective components of empathy (affective resonance and affective dissonance) is in line with studies reporting positive association between intense affect and vulnerability [51,52].

#### The role of narcissism–absence of direct inhibition

As in the female subsample, admiration was the chief driver of agency. However, in men both varieties of antagonistic narcissism (vulnerable narcissism and rivalry) only indirectly affected rebound. The strong inhibitory action of vulnerable narcissism on resilience was only indirect, mediated through agency, yet in agreement with our H1. Negative link between vulnerable narcissism and agency was stronger than the positive link between agency and admiration. This possibly indicates greater importance of avoidance strategies in stress enhancing fragility relative to strategies facilitating assertive and self-assured behavior as was reported by Horvath and Morf [53]. Through admiration, rivalry feebly gives support to agency, and consequently to resilience. Concurrently, rivalry strongly facilitates vulnerable narcissism thus indirectly inhibiting agency. However, as opposed to the female subsample, there was no direct influence of rivalry on resilience. Hence, absence of direct inhibitory effect of both types of narcissism on resilience was one of the key features of the male subsample. That is, gender-related variance in coping is established by the differences in avoidance strategies rather than by the differences in the proactive strategies. Seemingly, the absence of direct inhibitory strategies and the stronger impact of antagonism on dynamics of resilience were unique features of the male subsample. A recent study indicated that avoidance mechanism is associated with maladaptive personality traits [54]. Increased approach motivation and deficient inhibitory control predispose men for higher impulsivity relative to women [55]. Prosocial character of this mechanism is undeniable since there was no direct influence of antagonism on resilience. At the same time, males engage all dynamic personality resources that may lead to recovery, including those containing an admixture of antagonism.

### To what extent Machiavellianism and empathy activate resilience?

#### Machiavellianism and resilience

On the face of it, our finding on the central role of Machiavellianism in the structure of resilience should support the view expressed by Jones and Paulhus [56] that reduced reactivity to stress—caused by Machiavellian premeditation and caution—is among key benefits of Machiavellianism. Although Machiavellian agency was the key component in the resilience network in both genders, it is agentic [37], especially when its common variance with Machiavellian antagonism is partailed out. This finding about predominant protective role of agency somewhat contradicts recent reports on the chief protective role of antagonism in stress [32]. Secondly, expression of antagonism, the core feature of Machiavellianism was gender-related. In the female subsample antagonism exerted negligible, indirect, and negative influence on rebound. Antagonism’s importance for the network topology foremost involved strong suppression of the affective resonant response, which in turn played only a minor role in the network. In the male subsample, antagonism was the second strongest node of the network. Nevertheless, in the context of successful rebound, antagonism, when accompanied with affect, was redundant. The predominance of inhibitory mechanisms of resilience in women and the central role of proactive Machiavellianism in men is consistent with findings that emphasize the impact of gender identity and gender roles/socialization on one’s psychological well-being and coping strategies [57,58]. A meta-analysis examining gender and personality affirmed the notion that women generally exhibit a stronger communal orientation, whereas men tend to display higher agentic and instrumental tendencies. These distinctions remain relatively consistent across different generations and nations [59].

So, structural analysis upholds the necessity of multidimensional approach to Machiavellianism and can account for contradictory findings on predictive relationship between Machiavellianism and resilience.

#### Empathy and resilience

According to recent reports [52] and the biobehavioral synchronicity paradigm [3] empathy bolsters resilience. Surprisingly, our data suggest that empathy, and affective empathy in particular, plays only a secondary role in rebound from stress in both genders. Above all, we should keep in mind that in the present study resilience was operationalized as a behavioral variable, namely ‘the ability to bounce back or recover from stress’. It is quite plausible to assume that empathy sets up a general capacity for resilience and that rebound only reveals executive efficacy of this capacity. Some different operationalization of resilience—by use of characteristic coping mechanism, as a personality process, or as a presence/absence of personal wellbeing—would possibly sway our data in a different direction. That is, resilience defined as the product of complex interactions within the personality and personality’s complex interactions with its physical and social environment [1]. Empathy and resilience are the mainstays not only of individual personal development but also of the community as a whole [3]. Without mutual, synchronized action among individuals in immediate, and then indirect surroundings, there is no adaptability. This is why modern, multicultural societies [60] attach special importance to strategies that promote development of empathy as the foundation of cooperation and tolerance. Programs aimed at strengthening resilience, primarily directed towards children and adolescents, involve the development of empathy [61]. Ergo, “resilient community is based on a population of resilient people” [62].

### Limitations

As in most personality studies the data were obtained through self-reports. Since the official translations of the instruments were not used, the results must be taken with a reserve, e.g. results cannot be compared with the findings of other research done in the Serbian language. In future studies the use of official adaptations of the instruments is recommendable. Secondly, low reliability of Machiavellian planfulness threatens the overall interpretability of the data set in spite of satisfactory reliability of the remaining study variables. Thirdly, while the network analysis provided unique insights into topology of study variables, there are definite drawbacks which are inseparable from this methodology. Almost by definition, the network analysis operates with low coefficients of partial correlations; when applying the nonparametric bootstrap some confidence intervals entail zero which requires additional caution in data interpretation. Finally, resilience defined as a behavioral variable occupies the lowest level in the hierarchy of personality traits used in this study. Hence, there was no psychological rationale for interpretation of its redundancy in the female subsample. Furthermore, this manner of operationalization may account for low partial correlations between resilience and other study variables. In conclusion, the network perspective at the system of connected affective, cognitive and behavioral components may potentially alter our understanding of resilience.
